# Bis[bis­(1-oxo-2-pyrid­yl)­aminato]­copper(II) tetra­hydrate

**DOI:** 10.1107/S1600536809009453

**Published:** 2009-03-19

**Authors:** Liang-Gui Wang

**Affiliations:** a College of Chemistry and Life Science, Lishui University, 323000 Lishui, Zhejiang, People’s Republic of China

## Abstract

In the title compound, [Cu(C_10_H_8_N_3_O_2_)_2_]·4H_2_O, the Cu^II^ ion has a distorted octa­hedral coordination formed by four O [Cu—O = 2.051 (3)–2.083 (4) Å] and two N [Cu—N = 1.985 (4) and 1.996 (4) Å] atoms from two tridentate bis­(1-oxo-2-pyrid­yl)aminate ligands. In the two ligands, the pyridyl rings form dihedral angles of 21.0 (1) and 15.5 (1)°. The crystal packing exhibits an extensive network of O—H⋯O hydrogen bonds and π–π inter­actions proved by short distances of 3.650 (1) and 3.732 (2) Å between the centroids of pyridyl rings of neighbouring mol­ecules.

## Related literature

For general background, see Patra *et al.* (2004 ▶). For the crystal structures of related compounds, see: Kuang *et al.* (2006 ▶); Liu *et al.* (2007 ▶, 2008 ▶).
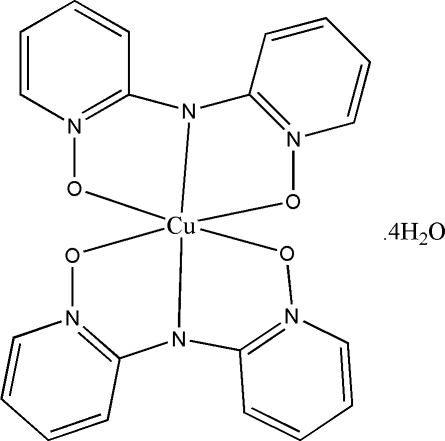


## Experimental

### 

#### Crystal data

[Cu(C_10_H_8_N_3_O_2_)_2_]·4H_2_O
*M* *_r_* = 539.99
Monoclinic, 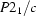

*a* = 10.697 (3) Å
*b* = 17.607 (5) Å
*c* = 15.052 (3) Å
β = 127.136 (14)°
*V* = 2260.0 (10) Å^3^
*Z* = 4
Mo *K*α radiation
μ = 1.03 mm^−1^
*T* = 298 K
0.29 × 0.22 × 0.18 mm

#### Data collection

Bruker APEXII area-detector diffractometer
Absorption correction: multi-scan (*SADABS*; Bruker, 2004 ▶) *T*_min_ = 0.755, *T*_max_ = 0.837
11476 measured reflections
4095 independent reflections
1752 reflections with *I* > 2σ(*I*)
*R*_int_ = 0.076

#### Refinement

*R*[*F* ^2^ > 2σ(*F* ^2^)] = 0.054
*wR*(*F* ^2^) = 0.159
*S* = 0.85
4095 reflections
340 parameters
12 restraints
H atoms treated by a mixture of independent and constrained refinement
Δρ_max_ = 0.60 e Å^−3^
Δρ_min_ = −0.46 e Å^−3^

### 

Data collection: *APEX2* (Bruker, 2004 ▶); cell refinement: *APEX2*; data reduction: *APEX2*; program(s) used to solve structure: *SHELXS97* (Sheldrick, 2008 ▶); program(s) used to refine structure: *SHELXL97* (Sheldrick, 2008 ▶); molecular graphics: *ORTEPIII* (Burnett & Johnson, 1996 ▶) and *ORTEP-3 for Windows* (Farrugia, 1997 ▶); software used to prepare material for publication: *SHELXL97*.

## Supplementary Material

Crystal structure: contains datablocks I, global. DOI: 10.1107/S1600536809009453/cv2529sup1.cif

Structure factors: contains datablocks I. DOI: 10.1107/S1600536809009453/cv2529Isup2.hkl

Additional supplementary materials:  crystallographic information; 3D view; checkCIF report

## Figures and Tables

**Table 1:** Hydrogen-bond geometry (Å, °)

*D*—H⋯*A*	*D*—H	H⋯*A*	*D*⋯*A*	*D*—H⋯*A*
O2*W*—H2*WA*⋯O3*W*^iii^	0.85 (7)	2.01 (7)	2.800 (7)	156 (6)
O3*W*—H3*WA*⋯O4	0.85 (3)	1.99 (5)	2.808 (6)	163 (7)
O2*W*—H2*WB*⋯O3	0.84 (6)	2.01 (6)	2.848 (6)	172 (8)
O1*W*—H1*WA*⋯O1^iv^	0.852 (10)	2.27 (4)	3.025 (6)	148 (7)
O1*W*—H1*WB*⋯O2*W*^ii^	0.86 (7)	1.96 (8)	2.755 (7)	153 (6)
O4*W*—H4*WB*⋯O4^v^	0.84 (3)	2.17 (3)	2.951 (6)	154 (6)

Symmetry codes: (ii) 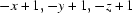; (iii) 
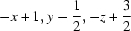
; (iv) 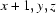; (v) 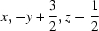.

**Fig. 1.**
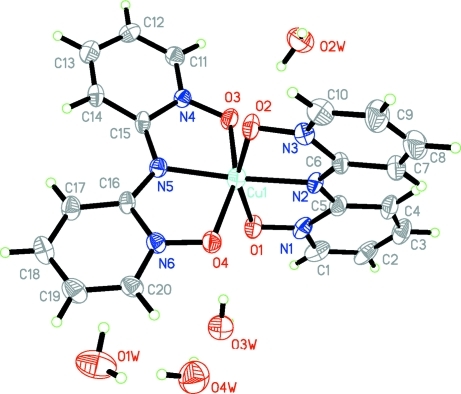
View of (I) showing the atom-labelling scheme and 30% probability displacement ellipsoids.

## supplementary crystallographic information

### Comment

Bis(amidopyridine) ligands have been widely explored in coordination chemistry for building various novel structural architectures and functional solid materials. Besides their diverse coordination modes, amide groups of ligands have proved to be useful in self-assembly, since they give predictable patterns of hydrogen bonding that can add extra dimensionality and helicity to the supramolecular structures (Patra *et al.*, 2004). The modified  bis(1-oxo-2-pyridyl)aminato ligand and its complexes have been recently reported (Liu *et al.*, 2007). In this paper, we report the synthesis and crystal structure of the title compound, (I).

The Cu^II^ atom in (I) (Fig.1) has a distorted octahedral coordination formed by two central N atoms and four O atoms of N-oxide groups from  two  bis(1-oxo-2-pyridyl)aminato ligands. This structure is very similar with [CuL_2_]^.^CH_3_OH compound (Kuang *et al.*, 2006). The average Cu—O bond length of 2.062 Å is close to the values observed in related complexes (Liu *et al.*, 2008).

The crystal packing exhibits  π–π interactions (Table 1) and an extensive network of O—H···O hydrogen bonds (Table 2).

### Experimental

Bis(1-oxo-2-pyridyl)aminato (0.062 g, 0.28 mmol), CuCl2 (0.024 g, 0.13 mmol), were added distilled water(12 mL), the mixture was heated for three hours under reflux. during the process stirring and influx were required. The resultant was kept at room temperature, three days later some single crystals of the size suitable for X-Ray diffraction measurement.

### Refinement

All H atoms attached to C atoms and N atom were fixed geometrically and treated as riding with C—H = 0.93 Å (benze ring) with U_iso_(H) = 1.2U_eq_(C or N). H atoms of the water molecules were located in difference Fourier maps and included in the subsequent refinement using  restraints (O—H =  0.85 (4) Å and H···H = 1.42 (4) Å), and treated as riding  with U_iso_(H) = 1.5U_eq_(O).

### Crystal data

**Table:**

[Cu(C_10_H_8_N_3_O_2_)_2_]·4H_2_O	*F*(000) = 1116
*M**_r_* = 539.99	*D*_x_ = 1.587 Mg m^−^^3^
Monoclinic, *P*2_1_/*c*	Mo *K*α radiation, λ = 0.71073 Å
Hall symbol: -P 2ybc	Cell parameters from 1752 reflections
*a* = 10.697 (3) Å	θ = 2.1–25.3°
*b* = 17.607 (5) Å	µ = 1.03 mm^−^^1^
*c* = 15.052 (3) Å	*T* = 298 K
β = 127.136 (14)°	Block, blue
*V* = 2260.0 (10) Å^3^	0.29 × 0.22 × 0.18 mm
*Z* = 4	

### Data collection

**Table:**

Bruker APEXII area-detector diffractometer	4095 independent reflections
Radiation source: fine-focus sealed tube	1752 reflections with *I* > 2σ(*I*)
graphite	*R*_int_ = 0.076
φ and ω scans	θ_max_ = 25.3°, θ_min_ = 2.1°
Absorption correction: multi-scan (*SADABS*; Bruker, 2004)	*h* = −10→12
*T*_min_ = 0.755, *T*_max_ = 0.837	*k* = −21→16
11476 measured reflections	*l* = −18→17

### Refinement

**Table:**

Refinement on *F*^2^	Primary atom site location: structure-invariant direct methods
Least-squares matrix: full	Secondary atom site location: difference Fourier map
*R*[*F*^2^ > 2σ(*F*^2^)] = 0.054	Hydrogen site location: inferred from neighbouring sites
*wR*(*F*^2^) = 0.159	H atoms treated by a mixture of independent and constrained refinement
*S* = 0.85	*w* = 1/[σ^2^(*F*_o_^2^) + (0.08*P*)^2^ + 0.2094*P*] where *P* = (*F*_o_^2^ + 2*F*_c_^2^)/3
4095 reflections	(Δ/σ)_max_ < 0.001
340 parameters	Δρ_max_ = 0.60 e Å^−^^3^
12 restraints	Δρ_min_ = −0.46 e Å^−^^3^

### Special details

**Table:**

Geometry. All esds (except the esd in the dihedral angle between two l.s. planes) are estimated using the full covariance matrix. The cell esds are taken into account individually in the estimation of esds in distances, angles and torsion angles; correlations between esds in cell parameters are only used when they are defined by crystal symmetry. An approximate (isotropic) treatment of cell esds is used for estimating esds involving l.s. planes.
Refinement. Refinement of F^2^ against ALL reflections. The weighted R-factor wR and goodness of fit S are based on F^2^, conventional R-factors R are based on F, with F set to zero for negative F^2^. The threshold expression of F^2^ > 2sigma(F^2^) is used only for calculating R-factors(gt) etc. and is not relevant to the choice of reflections for refinement. R-factors based on F^2^ are statistically about twice as large as those based on F, and R- factors based on ALL data will be even larger.

### Fractional atomic coordinates and isotropic or equivalent isotropic displacement parameters (Å^2^)

**Table:**

	*x*	*y*	*z*	*U*_iso_*/*U*_eq_	
Cu1	0.41065 (8)	0.62055 (4)	0.65999 (6)	0.0480 (3)	
O4	0.5914 (4)	0.6883 (2)	0.6952 (3)	0.0476 (10)	
O3	0.2092 (4)	0.5560 (2)	0.5640 (3)	0.0466 (10)	
O2	0.5541 (4)	0.5283 (2)	0.7419 (3)	0.0511 (10)	
O1	0.2746 (4)	0.7147 (2)	0.6342 (3)	0.0506 (10)	
N5	0.3971 (5)	0.6065 (2)	0.5228 (3)	0.0366 (11)	
N6	0.6029 (5)	0.6880 (2)	0.6100 (4)	0.0407 (11)	
N4	0.1976 (4)	0.5258 (2)	0.4770 (3)	0.0355 (10)	
N2	0.4349 (5)	0.6242 (3)	0.8015 (3)	0.0395 (11)	
C18	0.6318 (6)	0.6939 (4)	0.4418 (5)	0.0567 (17)	
H18	0.6409	0.6957	0.3841	0.068*	
N3	0.6122 (5)	0.5311 (3)	0.8499 (4)	0.0471 (12)	
N1	0.2569 (5)	0.7195 (3)	0.7140 (4)	0.0481 (13)	
C15	0.2965 (5)	0.5512 (3)	0.4521 (4)	0.0369 (13)	
C16	0.5016 (6)	0.6442 (3)	0.5173 (4)	0.0366 (14)	
C14	0.2811 (6)	0.5125 (3)	0.3655 (4)	0.0423 (14)	
H14	0.3488	0.5246	0.3483	0.051*	
C6	0.5502 (6)	0.5803 (3)	0.8852 (5)	0.0435 (14)	
C20	0.7135 (6)	0.7326 (3)	0.6203 (5)	0.0463 (15)	
H20	0.7788	0.7616	0.6844	0.056*	
C5	0.3413 (6)	0.6719 (3)	0.8065 (5)	0.0457 (15)	
C11	0.0881 (6)	0.4723 (3)	0.4162 (5)	0.0448 (14)	
H11	0.0214	0.4587	0.4338	0.054*	
C12	0.0727 (6)	0.4377 (3)	0.3297 (5)	0.0499 (15)	
H12	−0.0039	0.4010	0.2877	0.060*	
C17	0.5199 (6)	0.6495 (3)	0.4335 (5)	0.0497 (16)	
H17	0.4528	0.6216	0.3686	0.060*	
C4	0.3090 (7)	0.6789 (4)	0.8835 (5)	0.0543 (17)	
H4	0.3579	0.6463	0.9443	0.065*	
C2	0.1304 (7)	0.7794 (4)	0.7821 (6)	0.065 (2)	
H2	0.0616	0.8158	0.7745	0.078*	
C3	0.2077 (7)	0.7324 (4)	0.8717 (6)	0.0638 (19)	
H3	0.1914	0.7367	0.9257	0.077*	
C13	0.1727 (7)	0.4582 (4)	0.3055 (5)	0.0541 (16)	
H13	0.1656	0.4345	0.2473	0.065*	
C19	0.7303 (7)	0.7357 (4)	0.5374 (5)	0.0594 (18)	
H19	0.8080	0.7659	0.5455	0.071*	
C1	0.1547 (6)	0.7728 (3)	0.7021 (5)	0.0592 (18)	
H1	0.1016	0.8045	0.6401	0.071*	
C10	0.7378 (7)	0.4870 (4)	0.9224 (6)	0.0613 (18)	
H10	0.7791	0.4568	0.8951	0.074*	
C9	0.8059 (8)	0.4848 (4)	1.0328 (6)	0.074 (2)	
H9	0.8917	0.4537	1.0811	0.089*	
C8	0.7422 (8)	0.5307 (4)	1.0702 (6)	0.075 (2)	
H8	0.7840	0.5294	1.1452	0.091*	
C7	0.6194 (7)	0.5779 (4)	0.9999 (5)	0.0548 (16)	
H7	0.5807	0.6091	1.0281	0.066*	
O1W	0.9650 (6)	0.6775 (3)	0.4134 (4)	0.0916 (16)	
O2W	0.2615 (5)	0.4326 (3)	0.7051 (4)	0.0789 (14)	
O3W	0.4779 (6)	0.8374 (3)	0.6629 (5)	0.0815 (14)	
O4W	0.8955 (6)	0.8330 (4)	0.4159 (4)	0.0934 (16)	
H2WA	0.354 (4)	0.415 (4)	0.744 (6)	0.140*	
H2WB	0.250 (7)	0.472 (3)	0.669 (6)	0.140*	
H3WB	0.384 (4)	0.847 (4)	0.638 (7)	0.140*	
H1WA	1.029 (7)	0.684 (4)	0.4837 (14)	0.140*	
H1WB	0.911 (8)	0.637 (3)	0.398 (6)	0.140*	
H4WB	0.7981 (17)	0.832 (5)	0.365 (4)	0.140*	
H4WA	0.952 (6)	0.817 (5)	0.398 (6)	0.140*	
H3WA	0.499 (8)	0.7903 (12)	0.676 (7)	0.140*	

### Atomic displacement parameters (Å^2^)

**Table:**

	*U*^11^	*U*^22^	*U*^33^	*U*^12^	*U*^13^	*U*^23^
Cu1	0.0496 (4)	0.0564 (5)	0.0427 (5)	−0.0004 (4)	0.0303 (4)	−0.0027 (4)
O4	0.050 (2)	0.056 (3)	0.039 (2)	−0.010 (2)	0.0277 (19)	−0.005 (2)
O3	0.051 (2)	0.054 (3)	0.043 (2)	−0.0071 (19)	0.034 (2)	−0.011 (2)
O2	0.061 (2)	0.064 (3)	0.038 (2)	0.012 (2)	0.035 (2)	0.002 (2)
O1	0.056 (2)	0.060 (3)	0.040 (2)	0.010 (2)	0.032 (2)	0.002 (2)
N5	0.034 (2)	0.042 (3)	0.035 (3)	0.001 (2)	0.022 (2)	−0.001 (2)
N6	0.039 (3)	0.044 (3)	0.037 (3)	−0.001 (2)	0.022 (2)	0.004 (2)
N4	0.032 (2)	0.040 (3)	0.037 (3)	−0.001 (2)	0.022 (2)	−0.002 (2)
N2	0.044 (3)	0.047 (3)	0.034 (3)	0.004 (2)	0.027 (2)	−0.004 (2)
C18	0.046 (3)	0.081 (5)	0.048 (4)	0.004 (4)	0.031 (3)	0.013 (4)
N3	0.053 (3)	0.048 (3)	0.044 (3)	0.000 (3)	0.031 (3)	0.002 (3)
N1	0.037 (3)	0.062 (4)	0.041 (3)	−0.005 (3)	0.022 (2)	−0.017 (3)
C15	0.032 (3)	0.053 (4)	0.026 (3)	0.011 (3)	0.018 (3)	0.005 (3)
C16	0.029 (3)	0.053 (4)	0.026 (3)	0.004 (3)	0.016 (3)	0.003 (3)
C14	0.040 (3)	0.056 (4)	0.037 (3)	0.010 (3)	0.026 (3)	0.007 (3)
C6	0.052 (3)	0.051 (4)	0.040 (4)	−0.005 (3)	0.035 (3)	−0.002 (3)
C20	0.038 (3)	0.050 (4)	0.044 (4)	−0.008 (3)	0.021 (3)	0.001 (3)
C5	0.043 (3)	0.057 (4)	0.032 (3)	−0.012 (3)	0.020 (3)	−0.009 (3)
C11	0.041 (3)	0.047 (4)	0.050 (4)	−0.001 (3)	0.029 (3)	0.001 (3)
C12	0.048 (3)	0.045 (4)	0.049 (4)	−0.004 (3)	0.025 (3)	−0.010 (3)
C17	0.043 (3)	0.067 (4)	0.039 (4)	−0.004 (3)	0.024 (3)	−0.003 (3)
C4	0.054 (4)	0.075 (5)	0.043 (4)	−0.010 (4)	0.034 (3)	−0.014 (3)
C2	0.045 (4)	0.080 (5)	0.074 (5)	−0.006 (4)	0.037 (4)	−0.037 (4)
C3	0.054 (4)	0.094 (6)	0.057 (5)	−0.016 (4)	0.041 (4)	−0.025 (4)
C13	0.056 (4)	0.058 (4)	0.046 (4)	0.009 (4)	0.030 (3)	0.000 (3)
C19	0.044 (4)	0.070 (5)	0.061 (5)	−0.010 (3)	0.030 (4)	0.006 (4)
C1	0.039 (3)	0.056 (4)	0.064 (5)	0.001 (3)	0.021 (3)	−0.015 (3)
C10	0.054 (4)	0.061 (5)	0.065 (5)	0.009 (4)	0.033 (4)	0.009 (4)
C9	0.072 (4)	0.091 (6)	0.040 (4)	0.011 (4)	0.023 (4)	0.010 (4)
C8	0.082 (5)	0.081 (6)	0.044 (4)	−0.006 (5)	0.028 (4)	0.009 (4)
C7	0.064 (4)	0.063 (4)	0.041 (4)	−0.003 (4)	0.034 (3)	0.001 (3)
O1W	0.067 (3)	0.069 (4)	0.098 (4)	−0.002 (3)	0.029 (3)	0.017 (3)
O2W	0.087 (3)	0.069 (4)	0.082 (4)	−0.004 (3)	0.052 (3)	0.009 (3)
O3W	0.108 (4)	0.066 (3)	0.083 (4)	−0.003 (3)	0.064 (4)	−0.002 (3)
O4W	0.102 (4)	0.097 (4)	0.073 (4)	0.001 (4)	0.048 (3)	0.003 (3)

### Geometric parameters (Å, °)

**Table:**

Cu1—N2	1.985 (4)	C20—H20	0.9300
Cu1—N5	1.996 (4)	C5—C4	1.400 (7)
Cu1—O4	2.051 (3)	C11—C12	1.355 (7)
Cu1—O2	2.056 (4)	C11—H11	0.9300
Cu1—O3	2.064 (3)	C12—C13	1.371 (7)
Cu1—O1	2.083 (4)	C12—H12	0.9300
O4—N6	1.359 (5)	C17—H17	0.9300
O3—N4	1.348 (5)	C4—C3	1.364 (8)
O2—N3	1.347 (5)	C4—H4	0.9300
O1—N1	1.327 (5)	C2—C3	1.357 (9)
N5—C16	1.345 (6)	C2—C1	1.382 (8)
N5—C15	1.363 (6)	C2—H2	0.9300
N6—C20	1.350 (6)	C3—H3	0.9300
N6—C16	1.374 (6)	C13—H13	0.9300
N4—C11	1.342 (6)	C19—H19	0.9300
N4—C15	1.393 (6)	C1—H1	0.9300
N2—C5	1.343 (6)	C10—C9	1.354 (8)
N2—C6	1.353 (7)	C10—H10	0.9300
C18—C17	1.371 (7)	C9—C8	1.378 (9)
C18—C19	1.376 (8)	C9—H9	0.9300
C18—H18	0.9300	C8—C7	1.363 (8)
N3—C10	1.352 (7)	C8—H8	0.9300
N3—C6	1.377 (7)	C7—H7	0.9300
N1—C1	1.368 (7)	O1W—H1WA	0.852 (10)
N1—C5	1.393 (7)	O1W—H1WB	0.86 (7)
C15—C14	1.390 (7)	O2W—H2WA	0.85 (7)
C16—C17	1.391 (7)	O2W—H2WB	0.84 (6)
C14—C13	1.345 (7)	O3W—H3WB	0.85 (8)
C14—H14	0.9300	O3W—H3WA	0.85 (3)
C6—C7	1.410 (7)	O4W—H4WB	0.85 (8)
C20—C19	1.366 (7)	O4W—H4WA	0.85 (8)
			
Cg1···Cg3^i^	3.732 (2)	Cg2···Cg3^ii^	3.650 (1)
			
N2—Cu1—N5	174.15 (18)	N3—C6—C7	115.7 (5)
N2—Cu1—O4	102.22 (16)	N6—C20—C19	120.6 (5)
N5—Cu1—O4	78.90 (16)	N6—C20—H20	119.7
N2—Cu1—O2	79.40 (16)	C19—C20—H20	119.7
N5—Cu1—O2	94.82 (15)	N2—C5—N1	112.0 (5)
O4—Cu1—O2	93.35 (15)	N2—C5—C4	131.9 (6)
N2—Cu1—O3	100.02 (15)	N1—C5—C4	116.1 (5)
N5—Cu1—O3	79.34 (15)	N4—C11—C12	121.4 (5)
O4—Cu1—O3	157.50 (14)	N4—C11—H11	119.3
O2—Cu1—O3	94.08 (15)	C12—C11—H11	119.3
N2—Cu1—O1	78.36 (17)	C11—C12—C13	118.4 (5)
N5—Cu1—O1	107.41 (15)	C11—C12—H12	120.8
O4—Cu1—O1	91.64 (15)	C13—C12—H12	120.8
O2—Cu1—O1	157.76 (15)	C18—C17—C16	123.3 (5)
O3—Cu1—O1	89.47 (14)	C18—C17—H17	118.4
N6—O4—Cu1	111.2 (3)	C16—C17—H17	118.4
N4—O3—Cu1	110.0 (3)	C3—C4—C5	121.9 (6)
N3—O2—Cu1	109.1 (3)	C3—C4—H4	119.1
N1—O1—Cu1	110.3 (3)	C5—C4—H4	119.1
C16—N5—C15	126.8 (4)	C3—C2—C1	119.5 (6)
C16—N5—Cu1	117.3 (3)	C3—C2—H2	120.2
C15—N5—Cu1	115.2 (3)	C1—C2—H2	120.2
C20—N6—O4	117.4 (4)	C2—C3—C4	120.6 (6)
C20—N6—C16	122.8 (5)	C2—C3—H3	119.7
O4—N6—C16	119.8 (4)	C4—C3—H3	119.7
C11—N4—O3	118.1 (4)	C14—C13—C12	120.1 (6)
C11—N4—C15	122.3 (5)	C14—C13—H13	119.9
O3—N4—C15	119.7 (4)	C12—C13—H13	119.9
C5—N2—C6	126.6 (5)	C20—C19—C18	119.6 (6)
C5—N2—Cu1	117.5 (4)	C20—C19—H19	120.2
C6—N2—Cu1	115.8 (3)	C18—C19—H19	120.2
C17—C18—C19	118.4 (6)	N1—C1—C2	120.0 (6)
C17—C18—H18	120.8	N1—C1—H1	120.0
C19—C18—H18	120.8	C2—C1—H1	120.0
O2—N3—C10	117.5 (5)	N3—C10—C9	122.9 (6)
O2—N3—C6	120.7 (5)	N3—C10—H10	118.6
C10—N3—C6	121.7 (5)	C9—C10—H10	118.6
O1—N1—C1	117.6 (5)	C10—C9—C8	117.0 (7)
O1—N1—C5	120.5 (4)	C10—C9—H9	121.5
C1—N1—C5	121.9 (5)	C8—C9—H9	121.5
N5—C15—C14	132.4 (5)	C7—C8—C9	121.5 (6)
N5—C15—N4	112.9 (4)	C7—C8—H8	119.2
C14—C15—N4	114.5 (5)	C9—C8—H8	119.2
N5—C16—N6	112.9 (4)	C8—C7—C6	121.1 (6)
N5—C16—C17	131.8 (5)	C8—C7—H7	119.5
N6—C16—C17	115.3 (5)	C6—C7—H7	119.5
C13—C14—C15	123.1 (5)	H1WA—O1W—H1WB	111 (7)
C13—C14—H14	118.5	H2WA—O2W—H2WB	114 (8)
C15—C14—H14	118.5	H3WB—O3W—H3WA	112 (8)
N2—C6—N3	112.4 (5)	H4WB—O4W—H4WA	115 (3)
N2—C6—C7	131.8 (5)		

Symmetry codes: (i) *x*, −*y*−1/2, *z*−3/2; (ii) −*x*+1, −*y*+1, −*z*+1.

### Hydrogen-bond geometry (Å, °)

**Table:**

*D*—H···*A*	*D*—H	H···*A*	*D*···*A*	*D*—H···*A*
O2W—H2WA···O3W^iii^	0.85 (7)	2.01 (7)	2.800 (7)	156 (6)
O3W—H3WA···O4	0.85 (3)	1.99 (5)	2.808 (6)	163 (7)
O2W—H2WB···O3	0.84 (6)	2.01 (6)	2.848 (6)	172 (8)
O1W—H1WA···O1^iv^	0.85 (1)	2.27 (4)	3.025 (6)	148 (7)
O1W—H1WB···O2W^ii^	0.86 (7)	1.96 (8)	2.755 (7)	153 (6)
O4W—H4WB···O4^v^	0.84 (3)	2.17 (3)	2.951 (6)	154 (6)

Symmetry codes: (iii) −*x*+1, *y*−1/2, −*z*+3/2; (iv) *x*+1, *y*, *z*; (ii) −*x*+1, −*y*+1, −*z*+1; (v) *x*, −*y*+3/2, *z*−1/2.
